# Statin prescription patterns among patients with type 2 diabetes mellitus for primary and secondary prevention of cardiovascular disease

**DOI:** 10.1186/s12902-026-02242-w

**Published:** 2026-04-13

**Authors:** Asmaa Mohamed Abdelaal, Mohamed Ghonem, Yasmin Atwa Mohamed, Ahmad S. Hasan, Fady Kyrillos

**Affiliations:** 1 Department of Endocrinology and Diabetes (Internal medicine department), Faculty of Medicine, Mansoura University, Mansoura, Egypt; 2 Department of Clinical Pathology, Faculty of Medicine, Mansoura University, Mansoura, Egypt

**Keywords:** Statins prescription, Primary prevention, Secondary prevention, Cardiovascular risk, T2D

## Abstract

**Background:**

Individuals with Type 2 diabetes (T2D) face a significantly elevated cardiovascular disease (CVD) risk. Among the various risk factors, dyslipidemia is strongly associated with CVD. Statins are the first-line treatment for patients at risk. Their ability to effectively reduce low-density lipoprotein (LDL) levels has made them a cornerstone in the prevention and management of CVD.

**Aim:**

To assess the pattern of statin prescription in T2D patients and follow its use as a primary and secondary prevention for CVD.

**Patients and methods:**

The study design was cross-sectional followed by a quasi-experimental pre–post intervention. 210 T2D patients were recruited to evaluate the statin use regarding type, dose, adherence, and reason for use. After reporting the statin use pattern, all patients were subjected to one or more drug interventions: education about the importance of statin use, initiating statin in those who didn’t use it before, changing the statin type and/or dose, adding ezetimibe, and insisting on drug adherence. 3 months later, the statin use pattern was reevaluated to assess the percentage of patients who reached LDL and non high-density lipoprotein (non-HDL) targets according to 2023 European Society of Cardiology guidelines.

**Results:**

210 T2D patients were recruited in the study with a mean age of 54.63 ± 9.77 years. 67.6% were females, and 49% were at very high CV risk. Only 90 (42.9%) were statin users with 12.2% adherence rate. Atorvastatin was the most prescribed statin. 55.3% (57 out of 103) of patients with a very high CV risk and 48.6% (17 out of 35) of patients with high CV risk were not on statin therapy. High-intensity statins were used by only 28.2% and 31.4% of very high and high CV risk patients, respectively. A few patients achieved the LDL and non-HDL targets on the first visit, even among statin users (6.6% and 7.7%, respectively). Despite the increased percentage of target achievement on second visit, only 64.3% of all included patients achieved LDL target, and 57.6% achieved non-HDL target.

**Conclusion:**

The majority of T2D patients were either non-statin or moderate-intensity statin users, despite their high and very high CV risk. High-intensity statins plus ezetimibe cannot always achieve LDL and non-HDL targets. Potential need for additional lipid-lowering therapies should be addressed.

**Clinical trial number:**

Not applicable.

## Background

Type 2 diabetes (T2D) doubles the risk of cardiovascular diseases (CVD) and reduces life expectancy by 4–6 years [1]. CVD accounts for over 60% of deaths in diabetic patients [2]. Egypt, like other developing countries, is facing a high burden of CVD, and dyslipidemia is one of the most important modifiable risk factors for this burden [3].

Numerous clinical trials have demonstrated the beneficial impact of statin prescription on CVD outcomes [4]. According to 2025 American Diabetes Association (ADA) standards of medical care, statins were prescribed for the primary or secondary prevention of CVD [5]. Although the reduction of Low-density Lipoprotein (LDL) levels is the major effect of statins, many other pleiotropic effects have been suggested (anti-inflammatory and antioxidant effects) [6, 7].

According to 2023 European Society of Cardiology (ESC) Guidelines, LDL analysis is recommended as the primary lipid analysis method for screening, diagnosis, and management of dyslipidemia [8]. However, growing evidence suggests that non-High-density lipoprotein (non-HDL) is a stronger CVD predictor than LDL in diabetic patients [9].

Although the established role of statins in both primary and secondary prevention of CVD, several real-world studies have shown a distance between guidelines recommendations and the use of statin treatment [10, 11]. The benefits of statin therapy seen in randomized clinical trials will only be replicated in real life if patients adhere to the prescribed treatment regimen. But, about half of patients discontinue statin therapy within the first year, and adherence decreases with time. Patient, physician, and healthcare system-related factors play a role in this problem [12]. Treatment adverse effects are also a major source of low compliance, as 41% of patients discontinue statin therapy because of poor efficacy or adverse events [13]. Obtaining baseline data on patterns of statin prescriptions, prevalence, adherence to statin dosages, and sociodemographic and clinical determinants of statin prescription is critical for future interventions and prospective studies [14].

### Aim

To assess the statin prescription patterns for primary and secondary prevention of CVD, and follow statin use, compliance, and efficacy among patients with T2D.

## Subjects and methods

The study design was cross-sectional followed by a quasi-experimental pre–post intervention. 210 T2D patients were recruited from the outpatient clinics of Mansoura Specialized Medical Hospital, Mansoura University, Egypt. All participants were aged ≥ 30 years. Exclusion criteria included T1D, chronic liver disease, nephrotic syndrome, malignancy, immunocompromised patients, and pregnant women. Baseline sociodemographic data and diabetes details (diabetes duration, control, complications, antidiabetic medications, blood pressure assessment, and antihypertensive medications) were documented. The details of the statin used (reason for usage, type, dosage, side effects, and adherence) were reported. Statin adherence was evaluated by the patient’s self-reporting of his/her behavior towards the prescribed therapy. Patients were asked during the interview (1st visit) about the number of missed daily doses over the last 3 months. If the missed doses were above 20% of total doses over the previous 3 months, this was considered poor adherence.

Appropriate blood samples were taken from the included patients and sent to the biomedical laboratory in the hospital. Fasting plasma glucose levels, HbA1c, alanine transaminase (ALT), serum creatinine, total cholesterol, LDL, HDL, and triglycerides were analyzed via an automatic analyzer (AU640; Olympus, Japan). Non-HDL value was calculated by subtracting HDL from total cholesterol. Urine samples were collected to get the urinary albumin creatinine ratio for each patient. Glomerular filtration rate (eGFR) was estimated using Chronic Kidney Disease Epidemiology Collaboration (CKD-EPI) formula. CKD is defined as a reduction in eGFR 1.73 m2 to < 60 mL/min/ and/or persistent proteinuria (e.g. urinary albumin/creatinine ratio > 3 mg/mmol), sustained over ≥ 90 days [15].

The 2023 ESC cardiovascular risk stratification guideline [8] was applied to all included patients. LDL treatment goals were determined for every patient according to his/her CV risk; patients at very high risk required > 50% LDL reduction plus LDL of < 55 mg/dL, high-risk patients required > 50% LDL reduction plus LDL of < 70 mg/dL, and moderate risk patients required LDL of < 100 mg/dL. Targets of non-HDL are as follows: <85 mg/dL, < 100 mg/dL, and < 130 mg/dL for patients at very high, high, and moderate CV risk, respectively. LDL and non-HDL levels for every patient were compared with the recommended targets for each, according to 2023 ESC guidelines on CVD prevention. The recommended statin dose followed 2023 ESC guidelines. High-intensity statins (atorvastatin 40–80 mg, rosuvastatin 20–40 mg) for patients with diabetes at high or very high CV risk, as it lower LDL-C by 40–63%. Moderate-intensity statins (atorvastatin 10–20 mg, rosuvastatin 5–10 mg) for patients with diabetes at moderate CV risk, as it lower LDL-C by 30–49% [8].

After assessing the statin prescription patterns among the included patients, a quasi-experimental study (pre-post study) was performed. All patients were subjected to one or more of the following drug interventions; education about the importance of statin use, initiating statin therapy in those who didn’t use it before, changing the statin type and/or dose, adding ezetimibe, and insisting on drug adherence. Patients who achieved LDL and non-HDL targets on the 1st visit (according to CV risk and 2023 ESC guidelines) continued the same prescribed statins. Those who didn’t achieve the targets were prescribed high-intensity statins plus ezetimibe. 3 months later (2nd visit), total lipid profile and non-HDL levels for every patient were reanalyzed to assess the achievements of LDL and non-HDL targets. During this period, patient adherence to the prescribed statins was followed by one monthly phone call.

## Statistical analysis

Data analysis was performed by SPSS software, version 26 (SPSS Inc., PASW Statistics for Windows version 26. Chicago: SPSS Inc.). Qualitative data were described using numbers and percentages. Quantitative data were described using median (minimum and maximum) for non-normally distributed data and mean ± standard deviation for normally distributed data after testing normality using the Kolmogorov-Smirnov test. The significance of the results obtained was judged at the 0.05 level. Chi-squared test was used to compare qualitative data between two or more groups. The Mann-Whitney U test was used to compare the 2 studied groups for non-normally distributed data. The Wilcoxon signed-rank test was used to compare more than 2 periods studied. Student t-test was used to compare 2 independent groups for normally distributed data. Paired t-test was used to compare the 2 paired readings distributed data.

### Sample size

Sample size calculation was based on the pattern of statin prescription among diabetic cases retrieved from previous research [16]. Using Epi Info version 7.2.4.0 to calculate sample size based on 15.6% high-dose-intensity statins among participants who were at high risk of CVD, 95% CL with an acceptable margin of error = 5, then the total sample size will be 202 cases.

## Results

This study included 210 T2D patients with a mean age of 54.63 ± 9.77 years. 142 (67.6%) were females, and 103 (49%) were at very high CV risk. Other demographic and clinical data are demonstrated in Table 1. Laboratory findings are illustrated in Table 2.

**Table 1:** Demographic and clinical data of the patients studied

	*N* = 210	%
Age (years)		
Mean ± SD (min-max)	54.63 ± 9.77 (35–78)	
Gender		
Male/ Female	68/ 142	32.4/ 67.6
Residence		
Urban / Rural	85/ 125	40.5/ 59.5
Level of education		
None Primary school Secondary school College	99 17 45 49	47.1 8.1 21.4 23.3
Smoking	28	13.3
BMI (kg/m^2^) Mean ± SD (min-max)	33.65 ± 6.48 (21.9–56.0)	
Waist circumference (cm)		
Male Female	86.191 ± 14.17 84.7817 ± 15.2	
Duration of DM (years) Median (min-max)	9.0 (1week- 41.0 years)	
DM treatment		
Oral/ Insulin/ Both	99/ 92/ 19	47.1/ 43.8/ 9.0
Hypertension	140	66.7
Systolic blood pressure Mean ± SD (min-max)	125.33 ± 14.58 (90–160)	
Hypertension treatment None ARB ACEI CCB BB	*N* = 140 4 31 54 15 36	2.85 22.1 38.6 10.7 25.7
Family history of premature CVD death	38	18.1
CAD Stroke PAD	61 16 25	29.0 7.6 11.9
Retinopathy Neuropathy Nephropathy	77 196 67	36.7 93.3 31.9
ESC CV risk		
Moderate/ High/ Very high	72/ 35/ 103	34.3/ 16.7/ 49.0

BMI: body mass index, ARB: angiotensin receptor blockers, ACEI: angiotensin converting enzyme inhibitors, CCB: calcium channel blockers, BB: beta blockers, CVD: cardiovascular disease, CAD: coronary artery disease, PAD: peripheral arterial disease, ESC: European Society of Cardiology 2023, CV: cardiovascular

**Table 2:** Laboratory findings of the patients studied

HbA1c (%)	8.63 ± 2.18
Fasting plasma glucose (mg/dL)	236.7 ± 115.08
Creatinine (mg/dL)	1.11 ± 0.51
eGFR (mL/min/1.73m2)	76.04 ± 30.60
ALT (U/L)	18.25 ± 4.82
Albumin creatinine ratio (mg/g)	180 (1.5–4636)

eGFR: estimated glomerular filtration rate, ALT: alanine transaminase

Among the included patients (210), only 90 (42.9%) were using statins, with only 57 (63.3%) of them receiving the recommended doses, and only 11 (12.2%) were adherent to therapy. 81 (90%) of statin users were on monotherapy, with atorvastatin being the most prescribed statin (65.55%). High-intensity statin therapy was prescribed for 57 (63.33%) statin users, and all of them were non-adherent on medication. Financial constraint (53.2%) and lack of patient education (45.6%) were the main reasons for non-adherence. Only one patient reported myalgia as a cause for non-adherence (1.2%). Primary prevention was the main aim of statin therapy in this study (63.8%). The pattern of statin use in relation to CV risk is shown in Table 3. Most patients with high and very high CV risk weren’t on any statin therapy (48.6% and 55.3% respectively).

**Table 3:** Pattern of statin use according to CV risk stratification

1st Visit	ESC CV risk			Test of significance
Statin	Moderate *N* = 72	High *N* = 35	Very high *N* = 103	
Form None Monotherapy Combination	46 (63.9) 22 (30.6) 4 (5.6)	17 (48.6) 16 (45.7) 2 (5.7)	57 (55.3) 43 (41.7) 3 (2.9)	Mc = 3.90 *p* = 0.419
Dose Moderate intensity High intensity	*N* = 26 9 (34.6) 17(65.3)	*N* = 18 7 (38.8) 11(61.11)	*N* = 46 17 (36.95) 29(63.04)	ꭓ^2^=2.61 *p* = 0.625
Type Atorvastatin Rosuvastatin Rosuvastatin + ezetimibe Atorvastatin+ ezetimibe	*N* = 26 19 (73.07) 3 (11.5) 2 (7.6) 2 (7.6)	*N* = 18 11 (61.11) 5 (27.7) 2 (11.11) 0	*N* = 46 29 (63.04) 14 (30.43) 2 (4.3) 1 (2.2)	Mc = 8.34 *p* = 0.401

ESC: European Society of Cardiology, CV: cardiovascular, Mc: Monte Carlo test, ꭓ2: Chi-Square test, p: probability value

Regarding statin use, there were no significant differences in age, gender, residence, smoking, BMI, waist circumference, diabetes duration, or antidiabetic medications between statin users and non-users. Statin users showed a significantly higher prevalence of hypertension (77.8% vs. 58.3%, *p* = 0.003), and neuropathy (97.8% vs. 90%, *p* = 0.025), with lower HbA1c levels (8.22% vs. 8.95%, *p* = 0.016), compared to non-users. FPG levels, ALT, serum creatinine, eGFR, urine ACR ratio, and other micro- and macrovascular complications were comparable between the two groups. 86.9% of the male patients included in the study received the recommended statin dose, in comparison to 55.2% of the females. There was no significant difference between the recommended dose receivers and suboptimal dose receivers regarding age, residence, smoking, BMI, and WC.

Lipid profile parameters among statin users and non-users are shown in Table 4. There was no significant difference between statin users and non-users on first visit. However, both groups showed significant improvement in lipid profile on the 2nd visit. Table 5 demonstrates lipid parameters among recommended statin dose receivers and suboptimal dose receivers. There was no significant difference between both groups on the 1st visit. However, significant improvement in lipid profile was reported in both groups on the 2nd visit.

**Table 4:** Comparison of lipid profile levels between statin users and non-users on the 1st and 2nd visits

		Statin use		Test of significance
		No *N* = 120	Yes *N* = 90	
Cholesterol (mg/dl) Mean ± SD	First visit	202.97 ± 45.04	201.06 ± 55.39	t = 0.276 *p* = 0.783
	Second visit	149.15 ± 28.39	144.43 ± 30.24	t = 1.15 *p* = 0.248
Paired t-test, p-value		< 0.001*	< 0.001*	
TGs (mg/dl) Median (min-max)	First visit	149 (50–713)	149.5 (48–880)	z = 0.498 *p* = 0.618
	Second visit	100 (49–461)	100.5 (58–525)	z = 0.084 *p* = 0.933
Wilcoxon signed-rank test, p-value		< 0.001*	< 0.001*	
LDL (mg/dl) Mean ± SD	First visit	131.26 ± 34.72	123.89 ± 39.40	t = 1.44 *p* = 0.153
	Second visit	75.18 ± 19.42	71.34 ± 22.28	t = 1.33 *p* = 0.184
Paired t-test, p-value		< 0.001*	< 0.001*	
HDL (mg/dl) Mean ± SD	First visit	41.03 ± 13.04	41.92 ± 14.08	t = 0.475 *p* = 0.635
	Second visit	50.22 ± 12.56	48.48 ± 12.79	t = 0.985 *p* = 0.326
Paired t-test, p-value		*p* = 0.001*	*p* = 0.001*	
non-HDL (mg/dl) Mean ± SD	First visit	161.78 ± 43.42	159.41 ± 53.22	t = 0.355 *p* = 0.723
	Second visit	98.98 ± 23.76	96.41 ± 26.19	t = 0.743 *p* = 0.458
Paired t-test, p-value		*p* < 0.001*	*p* < 0.001*	

TGs: Triglycerides, t: Student t test, * statistically significant, Z: Mann-Whitney U test, p: probability value

**Table 5:** Comparison of different lipid profile levels according to the use of the recommended statin dose on the 1st and 2nd visits

		Recommended dose of Statin		Test of significance
		No *N* = 33	Yes *N* = 57	
Cholesterol Mean ± SD	First visit	202.36 ± 49.47	200.29 ± 58.96	t = 0.170 *p* = 0.866
	Second visit	139.75 ± 26.43	147.14 ± 32.15	t = 1.12 *p* = 0.267
Paired t test, p-value		t = 19.57 *p* < 0.001*	t = 12.92 *p* < 0.001*	
TGS Median (min-max)	First visit	149 (48–745)	150 (64–880)	z = 0.444 *p* = 0.657
	Second visit	94 (58–349)	101 (60–525)	z = 0.691 *p* = 0.490
Wilcoxon Signed rank test, p-value		*P* < 0.001*	*P* < 0.001*	
LDL Mean ± SD	First visit	124.73 ± 33.34	123.41 ± 42.79	t = 0.152 *p* = 0.880
	Second visit	67.75 ± 16.0	73.42 ± 25.11	t = 1.16 *p* = 0.246
Paired t test, p-value		*P* < 0.001*	*P* < 0.001*	
HDL Mean ± SD	First visit	43.05 ± 13.33	41.26 ± 14.56	t = 0.580 *p* = 0.564
	Second visit	48.61 ± 12.98	48.40 ± 12.81	t = 0.072 *p* = 0.943
Paired t test, p-value		0.023*	0.001*	
non-HDL Mean ± SD	First visit	160.22 ± 46.06	158.95 ± 57.34	t = 0.109 *p* = 0.914
	Second visit	91.36 ± 20.14	99.33 ± 28.89	t = 1.39 *p* = 0.165
Paired t test, p-value		< 0.001*	< 0.001*	

TGs: Triglycerides, t: Student t test, * statistically significant, Z: Mann-Whitney U test, p: probability value

The achievements of LDL and non-HDL targets on the 1st and 2nd visits among different subgroups are shown in Tables 6 and 7, and 8. Achievement of LDL and non-HDL targets was very low on the 1st visit, with no significant difference between either statin users and non-users or recommended dose receivers and suboptimal dose receivers. However, there was a significantly higher proportion of patients achieved LDL and non-HDL targets on the 2nd visit. LDL targets achievements were significantly higher among those with moderate CV risk versus high or very high CV risk patients. Unfortunately, not all patients achieved the targets on the 2nd visit even after using high intensity statin with ezetimibe combination and insisting on adherence.

**Table 6:** Achievement of LDL and non-HDL targets on the 1st and 2nd visits according to statin use

		Statin use		Test of significance
		No *N* = 120	Yes *N* = 90	
Target LDL n (%)	1st Visit	10 (8.3)	6 (6.6)	ꭓ^2^=0.203 *p* = 0.795
	2nd visit	76 (63)	59 (65.5)	t = 3.56 *p* = 0.739
McNemar test, p-value		*p* < 0.001*	*p* < 0.001*	
Target non-HDL n (%)	1st Visit	3 (2.5)	7 (7.77)	ꭓ^2^=3.159 *p* = 0.076
	2nd visit	65 (54.16)	56 (62.22)	ꭓ^2^=1.367 *p* = 0.242
McNemar test, p-value		*p* < 0.001*	*p* < 0.001*	

ꭓ2 = Chi-Square test, t: Student’s t test, p: probability value

**Table 7:** Achievement of LDL and non-HDL targets on the 1st and 2nd visits according to use of the recommended statin dose

		Recommended dose of statin		Test of significance
		No *N* = 33	Yes *N* = 57	
Target LDL n (%)	1st Visit	1 (3)	5 (8.8)	ꭓ^2^=1.11 *p* = 0.293
	2nd visit	24 (72.7)	35 (61.4)	ꭓ^2^=1.18 *p* = 0.276
McNemar test, p-value		0.001*	0.001*	
Target non-HDL n (%)	1st Visit	1 (3.0)	6 (10.5)	ꭓ^2^=1.64 *p* = 0.201
	2nd visit	23 (69.7)	33 (57.9)	ꭓ^2^=1.24 *p* = 0.367
McNemar test, p-value		0.001*	0.001*	

ꭓ2 = Chi-Square test, t: Student’s t test, p: probability value

**Table 8:** Achievement of LDL and non-HDL targets on the 1st and 2nd visits according to ESC CV risk stratification

		ESC CV risk			Test of significance
		Moderate *N* = 72	High *N* = 35	very high *N* = 103	
Target LDL n (%)	1st Visit	15 (20.8)	0	1 (1)	Mc = 27.22 *p* = 0.001*
	2nd visit	64 (88.9)	25 (71.4)	46 (44.7)	Mc = 37.04 *p* < 0.001*
McNemar test, p-value		*p* < 0.001*	*p* < 0.001*	*p* < 0.001*	
Target non-HDL n (%)	1st Visit	5 (6.9)	2 (5.7)	3 (2.9)	Mc = 1.60 *p* = 0.449
	2nd visit	38 (52.8)	28 (80)	55 (53.4)	Mc = 8.62 *p* = 0.013*
McNemar test, p-value		*p* < 0.001*	*p* < 0.001*	*p* < 0.001*	
Percentage of missed doses on 2nd visit (%) Median (min-max)		7 (1–18)	7 (1–13)	10 (1–18)	KW = 11.44 *p* = 0.003*

ESC: European Society of Cardiology, CV: cardiovascular, MC: Monte Carlo test, KW: Kruskal-Wallis, p: probability value

## Discussion

Among the included patients (210), only 90 (42.9%) were using statins, with only 57 (63.3%) of them receiving the guidelines recommended doses, and only 11 (12.2%) were adherent to the prescribed statins. None of the adherent patients were taking the guidelines recommended doses. Financial constraints (53.2%) and lack of patient education (45.6%) were the main reasons for non-adherence, suggesting the need for more patient education, financial support programs, polypill strategies, nurse-led lipid clinics, and pharmacist involvement. 81 (90%) of statin users were on monotherapy, with atorvastatin being the most prescribed statin (65.55%). The statin use was mainly for primary prevention (63.8%). Previous studies reported different percentages of statin users among diabetic subjects. Consistent with our study, two previous studies conducted in Ethiopia reported the same low percentage of statin users among diabetic subjects: 36.67% [17], and 55.7% [18], with lovastatin and simvastatin being the most frequently prescribed statins in these 2 studies, respectively. However, a study in Malaysia, which included 576 T2D patients, showed that 81.1% of patients were statin users, with an adherence rate of 56.5%, and atorvastatin was the most prescribed statin [19]. Another cross-sectional study conducted in Saudi Arabia on 167 diabetic patients revealed that statin users were 122 patients (73.1%). 25.4% of patients were non-adherent, with myalgia the most common cause, followed by elevated liver enzymes. Rosuvastatin was the most prescribed statin, followed by atorvastatin and simvastatin [20].

Statin intolerance in our study was reported in only one patient out of ninety of statin users on the 1st visit (1.2%). This patient reported myalgia and was using atorvastatin 80 mg/day. Statin intolerance was infrequent (1–5%) in previous randomized controlled trials, and more frequent (5–10%) in observational studies and in clinical practice [21].

Our results showed that 55.3% (57 out of 103) of patients with a very high CV risk and 48.6% (17 out of 35) of patients with high CV risk were not on statin therapy. High-intensity statins were used by only 28.2% and 31.4% of very high and high CV risk patients, respectively. This doesn’t align with the guidelines recommending high-intensity statins for all patients at high and very high CV risk [8]. This remarkable undertreatment may be due to false physician-perceived CV risk. A recent study reported a significant discrepancy between physician-perceived and calculated CV risk, leading to risk underestimation in over one-third of patients. This underestimation was associated with lower LDL target achievement and reduced statin use [22].

Aligned with our results, ***De Backer et al.*** found that only 49.9% of very high-risk patients were treated with high-intensity statins, and only 2.7% received combination therapy with statin and ezetimibe [23]. Also, ***Ray et al.*** revealed that high-intensity statin was prescribed to only 20% of very high CV risk patients in primary prevention and 37.5% in secondary prevention [11].

The current study showed that only 1% of the very high-risk patients and none of the high-risk patients reached the LDL target on the first visit. Non-HDL target was reached in only 2.9% of the very high-risk patients and 5.7% of high-risk patients. Even after ensuring the use of recommended statin dose plus ezetimibe and ensuring adherence on the second visit, not all patients with very high and high CV risk achieved the targets. LDL target was achieved in only 44.7% of very high-risk and 71.4% of high-risk patients. Non-HDL target was achieved in only 53.4% of very high-risk and 80% of high-risk patients. In accordance with our results, multiple previous studies reported a low percentage of achieving the LDL targets among the very high CV risk patients: 22% [11], 36.6% [23], 28.1% [24, 25].

Our results reported no significant differences regarding age, gender, residence, smoking status, BMI, or waist circumference between statin users and non-users, on the first visit. In agreement, two previous studies reported the same findings [17, 26]. This suggests that statin prescription patterns were not influenced by these demographic factors, but rather by clinical indications such as CV risk.

The current study showed that statin users had a significantly higher prevalence of hypertension (77.8% vs. 58.3%) and neuropathy (97.8% vs. 90%) compared to non-users. This indicates that statins were more likely to be prescribed to patients with additional CV risk factors or complications. The higher prevalence of hypertension in statin users aligns with guidelines recommending statins for patients with hypertension and T2D. These findings are consistent with two previous studies, which revealed that hypertension was positively associated with statin use in T2D patients [14, 16]. In disagreement with our results, one study reported that statin users had a significantly lower prevalence of hypertension (48.4% vs. 74.2%) [20]. Another study reported no association between statin use and hypertension or neuropathy [18].

Statin users in the current study had significantly lower HbA1c levels compared to non-users (8.22% vs. 8.95%, respectively) (*p* = 0.016), suggesting better glycemic control in this group. Some studies reported the same finding [18, 20], others reported no significant difference between the 2 groups regarding HbA1c [14, 26].

Lipid profile parameters were comparable between statin users and non-users on the first visit of the current study. This may be due to a lack of adherence, using the non-recommended doses, or the need to use lipid-lowering drug combinations. However, both statin users and previously non-users showed significant reductions in cholesterol, triglyceride, LDL, and non-HDL levels on the second visit compared to the first visit, with a significant increase in HDL levels. This significant change was after initiating statin in the non-users’ group, adjusting the dose to high-intensity statin and/or adding ezetimibe, and ensuring adherence in both groups. Following our results, ***Bideberi & Mutagaywa*** reported that there was no significant difference between statin users and non-users regarding LDL levels [14]. Contrary to our results, ***Ismail et al.*** reported that there was a significant difference between statin users and non-users regarding cholesterol, triglyceride, LDL, and HDL levels [20].

The current study showed no significant difference between statin users and non-users regarding achieving LDL and non-HDL targets on the 1st visit. However, there was a significantly higher proportion of patients who achieved these targets on the 2nd visit. On the 2nd visit, 64.3% of all included patients achieved LDL targets, while 57.6% achieved non-HDL targets. Unfortunately, not all patients reached the targets on the 2nd visit, even after ensuring adherence to high intensity statin plus ezetimibe. This finding can be explained by the very high baseline CV risk and the relatively short follow-up duration. This also might indicate the potential need for additional lipid-lowering therapies (e.g., proprotein convertase subtilisin/kexin type 9 (PCSK9) inhibitors or bempedoic acid) [27]. In agreement with our results, a single-center study in Belgium reported that only 63.2% of patients reached LDL-C target of < 55 mg/dL after one year of treatment with high-intensity statin therapy [25]. Another multicenter study in Saudi Arabia also reported that only 49.2% of statin users met these targets [28].

The current study showed that 86.9% of the included male patients were receiving the guidelines recommended statin doses, in comparison to 55.2% of the females. This suggests that gender may influence statin dosing, but further investigations are needed to understand the underlying reasons. Aligned with our results, ***Bideberi & Mutagaywa*** reported that patients who received recommended statin doses were less likely to be female [14].

***Hafiz et al.*** reported that LDL levels were significantly lower in patients receiving the guidelines recommended statin doses versus suboptimal dose receivers. In contrast, Lipid profile parameters were comparable in patients receiving the recommended statin dose and suboptimal dose on the 1st visit of the current study [28]. This may be due to lack of adherence or the need for lipid-lowering combination therapy. However, both groups showed significant reductions in total cholesterol, triglyceride, LDL, and non-HDL levels on the 2nd visit, with a significant increase in HDL levels. This was achieved after ensuring adherence and adjusting the dose to a high-intensity statin plus ezetimibe.

The current study showed no significant difference between receivers of the recommended statin dose and suboptimal dose receivers, regarding achieving the LDL and non-HDL targets on the 1st visit. Non-adherence can explain this finding. However, there was a significantly higher proportion of patients who achieved targets on the 2nd visit. On the 2nd visit, LDL and non-HDL targets were achieved in 59 (65.55%) and 56 (62.22%) out of 90 patients, respectively. Unfortunately, not all patients reached the targets on the 2nd visit, even after ensuring adherence and use of the recommended dose plus ezetimibe. It might indicate the need for adding other lipid-lowering drugs [27]. Corresponding to our results, previous studies reported that only 50% of the patients receiving the recommended statin doses achieved LDL targets at follow-up [28, 29].

## Study strength

The current study addresses a highly relevant issue in the comprehensive care of people living with diabetes. It highlights the gaps in statin prescribing practice among clinicians and adherence among patients with a high CV burden, in real-world clinical practice. The interventional nature of study, rather than just observational, is one of the main strengths. In comparison, the previous larger multicenter trials conducted in Egypt, like DYSIS-Egypt study [24], CEPHEUS I [30], and CEPHEUS II [31] were observational, retrospective single-visit trials. Another strength of the current study is that it uses the current guidelines (ESC 2023) for risk stratification and therapeutic target definition. It also included a full detailed history and patient examination to categorize these patients according to their CV risk. The current study is also considered as one of the few studies that highlighted the importance of non-HDL target al.ongside LDL among diabetic subjects.

## Study limitations

The relatively small sample size and the single health facility in which the study was conducted substantially limiting external validity. However, intervention included multiple components (education, initiation or intensification of statins, addition of ezetimibe, and adherence monitoring), the study didn’t determine the relative contribution of each component. The sample size and event rate was insufficient to support a robust multivariable model to evaluate whether specific clinical or therapeutic variables influence the probability of achieving lipid targets. More studies are needed for adjustment of confounding factors and provide clinically meaningful insights into which variables independently predict treatment success in real-world practice. Drug adherence in the current study was assessed by patients self-reporting through one monthly phone call. Future studies should use validated adherence tools. Another limitation is the relatively short follow-up duration (3 months) for assessing sustained adherence or true lipid-lowering treatment efficacy. New treatment options for lipid-lowering therapy like PCSK9 inhibitors or bempedoic acid were not evaluated in our study due to the high cost and/ or unavailability. More studies are needed to address the newer lipid-lowering therapies with longer follow-up durations.

## Conclusion

The majority of T2D patients were non-statin users despite the high and very high CV risk. Discrepancy between guideline recommendations and practice was also obviously noted regarding statin dose intensity, drug combinations, and adherence. Although physicians are aware of the general concept of CV risk, they fail to consistently implement guidelines. Despite initiating statins and insisting on adherence to high-intensity doses plus ezetimibe, the achievement of LDL and non-HDL targets was not satisfactory. Potential need for additional lipid-lowering therapies (e.g., Bempedoic acid and PCSK9 inhibitors) should be addressed. This finding is alarming and is a call for action to improve the execution of clinical practice guidelines for the benefit of this high-risk population.

## Data Availability

The original contributions presented in the study are included in the article; further inquiries can be directed to the corresponding author.

## Abbreviations

T2D: Type 2 diabetes
T1D: Type 1 diabetes
CVD: Cardiovascular disease
LDL: Low Density Lipoprotein
HDL: High Density Lipoprotein
TG: Triglycerides
PAD: Peripheral Arterial Disease
ALT: Alanine Transaminase
ADA: American Diabetes Association
ESC: European Society of Cardiology

## References

[1] Sattar N, Rawshani A, Franzén S, Rawshani A, Svensson AM, Rosengren A, McGuire DK, Eliasson B, Gudbjörnsdottir S. Age at diagnosis of type 2 diabetes mellitus and associations with cardiovascular and mortality risks: findings from the Swedish National Diabetes Registry. Circulation. 2019;139(19):2228–37. 30955347 10.1161/CIRCULATIONAHA.118.037885

[2] Gaidai O, Cao Y, Loginov S. Global cardiovascular diseases death rate prediction. Curr Probl Cardiol. 2023;48(5):101622. 36724816 10.1016/j.cpcardiol.2023.101622

[3] Reda A, Ragy H, Saeed K, Alhussaini MA. A semi-systematic review on hypertension and dyslipidemia care in Egypt—highlighting evidence gaps and recommendations for better patient outcomes. J Egypt Public Health Assoc. 2021;96(1):32. 34851468 10.1186/s42506-021-00096-9 PMC8634749

[4] Chou R, Cantor A, Dana T, Wagner J, Ahmed AY, Fu R, Ferencik M. Statin use for the primary prevention of cardiovascular disease in adults: updated evidence report and systematic review for the US Preventive Services Task Force. JAMA. 2022;328(8):754–71. 35997724 10.1001/jama.2022.12138

[5] American Diabetes Association Primary Care Advisory Group. Standards of Care in Diabetes—2025 Abridged for Primary Care. Clin Diabetes 18 April. 2025;43(2):182. 10.2337/cd25-aint PMC12018997 40290834

[6] Oesterle A, Laufs U, Liao JK. Pleiotropic effects of statins on the cardiovascular system. Circul Res. 2017;120(1):229–43. 10.1161/CIRCRESAHA.116.308537 PMC5467317 28057795

[7] Mortensen MB, Falk E, Li D, Nasir K, Blaha MJ, Sandfort V, Rodriguez CJ, Ouyang P, Budoff M. Statin trials, cardiovascular events, and coronary artery calcification: implications for a trial-based approach to statin therapy in MESA. JACC: Cardiovasc Imaging. 2018;11(2 Part 1):221–30. 10.1016/j.jcmg.2017.01.029 PMC5723240 28624395

[8] Marx N, Federici M, Schuett K, Mueller-Wieland D, Ajjan RA, Antunes MJ, Christodorescu RM, Crawford C, Di Angelantonio E, Eliasson B, Espinola-Klein C. 2023 ESC Guidelines for the management of cardiovascular disease in patients with diabetes: Developed by the task force on the management of cardiovascular disease in patients with diabetes of the European Society of Cardiology (ESC). Eur Heart J. 2023;44(39):4043–140. 37622663 10.1093/eurheartj/ehad192

[9] Akbar M, Jha D, Ali H, Ahmad T. Non-HDL estimation methods: advancing cardiovascular disease prediction. Indian J Pharm Pract. 2023;16(4).

[10] Morieri ML, Perrone V, Veronesi C, Degli Esposti L, Andretta M, Plebani M, Fadini GP, Vigili de Kreutzenberg S, Avogaro A. Improving statin treatment strategies to reduce LDL-cholesterol: factors associated with targets’ attainment in subjects with and without type 2 diabetes. Cardiovasc Diabetol. 2021;20(1):144. 34271920 10.1186/s12933-021-01338-y PMC8283985

[11] Ray KK, Molemans B, Schoonen WM, Giovas P, Bray S, Kiru G, Murphy J, Banach M, De Servi S, Gaita D, Gouni-Berthold I. EU-Wi de Cross-Section al Obser v at ion al Study of Lipid-Modifying Therapy Use in Se c ondary and Pr i mary Care: the DA VINCI study. Eur J Prev Cardiol. 2021;28(11):1279–89. 33580789 10.1093/eurjpc/zwaa047

[12] Maningat P, Gordon BR, Breslow JL. How do we improve patient compliance and adherence to long-term statin therapy? Curr Atheroscler Rep. 2013;15(1):291. 23225173 10.1007/s11883-012-0291-7 PMC3534845

[13] Banach M, Penson PE. Adherence to statin therapy: it seems we know everything, yet we do nothing. Eur heart J open. 2022;2(6):oeac071. 36440352 10.1093/ehjopen/oeac071 PMC9683386

[14] Bideberi AT, Mutagaywa R. Statin prescription patterns and associated factors among patients with type 2 diabetes mellitus attending diabetic clinic at Muhimbili National Hospital, Dar es Salaam, Tanzania. Diabetes, metabolic syndrome and obesity: targets and therapy. 2022 Jan 1:633–46. 10.2147/DMSO.S347765 PMC8894101 35250285

[15] Navaneethan SD, Zoungas S, Caramori ML, Chan JC, Heerspink HJ, Hurst C, Liew A, Michos ED, Olowu WA, Sadusky T, Tandon N. Diabetes management in chronic kidney disease: synopsis of the KDIGO 2022 clinical practice guideline update. Ann Intern Med. 2023;176(3):381–7. 36623286 10.7326/M22-2904

[16] Nigussie S, Demeke F. Prescribing patterns of statins and associated factors among type 2 diabetes mellitus patients attended at Jugol General Hospital in eastern Ethiopia: A cross-sectional study. Front Clin diabetes Healthc. 2023;4:1061628. 37034477 10.3389/fcdhc.2023.1061628 PMC10076854

[17] Melaku T, Solomon Y, Chelkeba L. Statin utilization patterns among Type 2 diabetes mellitus patients with high cardiovascular disease risks in Ethiopia. J Pharm Care. 2018:44–51.

[18] Demoz GT, Wahdey S, Kasahun GG, Hagazy K, Kinfe DG, Tasew H, Bahrey D, Niriayo YL. Prescribing pattern of statins for primary prevention of cardiovascular diseases in patients with type 2 diabetes: insights from Ethiopia. BMC Res Notes. 2019;12(1):386. 31288848 10.1186/s13104-019-4423-9 PMC6617647

[19] Hammad MA, Sulaiman SA, Aziz NA, Noor DA. Prescribing statins among patients with type 2 diabetes: The clinical gap between the guidelines and practice. J Res Med Sci. 2019;24(1):15. 30988683 10.4103/jrms.JRMS_100_18 PMC6421885

[20] Ismail HM, Shora HA, Al-Nozha O, Yamany HA, Al-Nozha F, Zaitone SA, Alahmdi H, Aljohani A, Almohammadi N, Abdallah AR. Pattern of Statin use in patients with diabetes mellitus type 2 for primary and secondary prevention of cardiovascular disease in Saudi Arabia. Afr J Diabetes Med. 2020;28(2).

[21] Grundy SM, Stone NJ, Bailey AL, Beam C, Birtcher KK, Blumenthal RS, Braun LT, de Ferranti S, Faiella-Tommasino J, Forman DE, et al. 2018 AHA/ACC/AACVPR/AAPA/ABC/ACPM/ADA/AGS/APhA/ASPC/NLA/PCNA. Guideline on the Management of Blood Cholesterol: A Report of the American College of Cardiology/American Heart Association Task Force on Clinical Practice Guidelines. Circulation. 2019;139:e1082–143. 10.1161/CIR.0000000000000625 PMC7403606 30586774

[22] Cesaro A, Acerbo V, Scialla F, Golia E, Concilio C, Scherillo G, De Michele G, de Sio V, Capolongo A, Di Donato L, Monaco MG. Discrepancies between physician-perceived and calculated cardiovascular risk in primary prevention: implications for LDL-C target achievement and appropriate lipid-lowering therapy. High Blood Press Cardiovasc Prev. 2025;32(2):199–208. 39969794 10.1007/s40292-025-00705-0 PMC11890243

[23] De Backer G, Jankowski P, Kotseva K, Mirrakhimov E, Reiner Ž, Ryden L, Tokgözoğlu L, Wood D, De Bacquer D, De Backer G, Jankowski P. Management of dyslipidaemia in patients with coronary heart disease: results from the ESC-EORP EUROASPIRE V survey in 27 countries. Atherosclerosis. 2019;285:135–46. 31054483 10.1016/j.atherosclerosis.2019.03.014

[24] El Etriby A, Bramlage P, El Nashar A, Brudi P, DYSIS Egypt Investigators. The DYSlipidemia International Study (DYSIS)-Egypt: a report on the prevalence of lipid abnormalities in Egyptian patients on chronic statin treatment. Egypt Heart J. 2013;65(3):223–32.

[25] Henry JP, Gabriel L, Luchian ML, Higny J, Benoit M, Xhaët O, Blommaert D, Telbis AM, Robaye B, Guedes A, Demeure F. Evaluating the Efficacy of a Pre-Established Lipid-Lowering Algorithm in Managing Hypercholesterolemia in Patients at Very High Cardiovascular Risk. J personalized Med. 2024;14(10):1044. 10.3390/jpm14101044 PMC11509033 39452551

[26] Mwita JC, Godman B, Esterhuizen TM. Statin prescription among patients with type 2 diabetes in Botswana: findings and implications. BMC Endocr disorders. 2020;20(1):36. 10.1186/s12902-020-0516-7 PMC7063760 32151249

[27] Scicali R, Di Pino A, Ferrara V, Urbano F, Piro S, Rabuazzo AM, Purrello F. New treatment options for lipid-lowering therapy in subjects with type 2 diabetes. Acta Diabetol. 2018;55(3):209–18. 29260404 10.1007/s00592-017-1089-4

[28] Hafiz A, Aljohani S, Kutbi H, Fatani N, Alkhathran L, Alyaqub M, Alhamed MS, Alhaqbani AO, Alhadlaq AA, Alsalman MA, Al Yami MS. Statin Use and Major Adverse Cardiovascular Events Among Patients with Ischemic Heart Diseases: A Multi-Center Retrospective Study. J Clin Med. 2025;14(3):908. 39941579 10.3390/jcm14030908 PMC11818335

[29] Jyotsna FN, Ahmed A, Kumar K, Kaur P, Chaudhary MH, Kumar S, Khan E, Khanam B, Shah SU, Varrassi G, Khatri M. Exploring the complex connection between diabetes and cardiovascular disease: analyzing approaches to mitigate cardiovascular risk in patients with diabetes. Cureus. 2023;15(8). 10.7759/cureus.43882 PMC10511351 37746454

[30] Reda A, Abdel-Rehim AA, Etman A, Afifi OSA. Centralized Pan-Middle East Survey on the under-treatment of hypercholesterolemia: results from the CEPHEUS Study in Egypt. Cardiol Ther apy. 2014;3(1–2):27–40. 10.1007/s40119-014-0031-x PMC4265229 25403341

[31] Reda A, Etman A, Abdel-Rahim A, Farag N, Sanad O, Salamah S. Centralized Pan-Middle East survey on the under-treatment of hypercholesterolemia: results from the CEPHEUS II study in Egypt. Cardiol Therapy. 2017;6(1):105–20. 10.1007/s40119-017-0089-3 PMC5446822 28357773

